# Impact of germline *BRCA1/2* mutations on response to neoadjuvant systemic therapy and prognosis in breast cancer: a propensity score matched cohort study

**DOI:** 10.1186/s13058-025-02041-6

**Published:** 2025-05-22

**Authors:** Hyunyou Kim, Jung Whan Chun, Jinha Hwang, Seung Gyu Yun, Jinseob Kim, Seung Pil Jung, Hyeong-Gon Moon, Eun-Shin Lee, Wonshik Han

**Affiliations:** 1 Department of Surgery, Korea University Hospital, Korea University College of Medicine, Seoul, Republic of Korea; 2 Department of Surgery, Seoul National University College of Medicine, Seoul, Republic of Korea; 3 Department of Laboratory Medicine, Korea University Hospital, Korea University College of Medicine, Seoul, Republic of Korea; 4 Zarathu Co., Ltd, Seoul, Republic of Korea; 5 Division of Breast-Endocrine Surgery, Department of Surgery, Korea University Anam Hospital, Seoul, Korea

**Keywords:** Breast cancer, *BRCA1/2* mutation, Neoadjuvant chemotherapy, Pathologic complete response, Distant metastasis-free survival

## Abstract

**Background:**

We investigated whether germline *BRCA1/2* pathogenic variants (PVs) influence treatment response and survival outcomes in breast cancer patients treated with neoadjuvant chemotherapy (NCT). Using propensity score matching (PSM) to control for variations in treatment and clinicopathological characteristics, this study aimed to evaluate the influence of *BRCA1/2* mutations on prognosis and treatment efficacy, providing insights for optimizing therapeutic strategies and improving patient outcomes.

**Methods:**

We conducted a retrospective cohort study using data from two institutions. The study analyzed breast cancer patients who underwent germline *BRCA1/2* testing and received NCT followed by curative resection and standard adjuvant therapy from January 2001 to January 2019. PSM was used to balance confounding variables.

**Results:**

Among 411 patients included, 86 have *BRCA1/2* mutations. After matching, *BRCA1/2* PV carriers had a higher pCR rate (40.0%) compared to wild-type patients (26.5%, OR = 1.85, 95% CI: 1.07–3.22, *P =* 0.029). They also exhibited a significantly lower 5-year DM rate (4.7% vs. 18.2%, OR = 0.22, 95% CI: 0.08–0.65, *P =* 0.006). Among pCR patients, outcomes were excellent regardless of *BRCA1/2* status. For non-pCR patients, *BRCA1/2* PV carriers had better DMFS (hazard ratio (HR) = 0.27, 95% confidence interval (CI) = 0.09–0.81, *P =* 0.02), though overall survival differences were not significant (HR = 0.47, 95% CI = 0.15–1.47, *P =* 0.197).

**Conclusions and relevance:**

Germline *BRCA1/2* mutations are associated with higher pCR rates and improved DMFS in breast cancer patients treated with NCT. These findings emphasize the enhanced chemosensitivity of *BRCA*-associated tumors and the importance of genetic testing in treatment planning. Further research is needed to validate these findings and optimize treatment strategies.

**Supplementary Information:**

The online version contains supplementary material available at 10.1186/s13058-025-02041-6.

## Introduction

*BRCA1* and *BRCA2* genes (*BRCA1/2*) are extensively studied for their role in germline predisposition associated with hereditary breast and ovarian cancer syndrome [1–8]. Deleterious mutations in *BRCA1* or *BRCA2* lead to defects in DNA damage repair due to homologous recombination deficiency, resulting in a high penetrance for both breast and ovarian cancer following an autosomal-dominant inheritance pattern [9–12]. Research has identified several key characteristics of germline *BRCA1/2* pathogenic variants (PVs) in relation to breast cancer. Patients with *BRCA1/2* mutations have a significantly higher lifetime risk of developing breast cancer and are more likely to experience bilateral breast cancer and an earlier onset of the disease [13–16].

Previous studies have shown contradictory findings about breast cancer-specific mortality between *BRCA1/2* PV carriers and noncarriers. While some studies reported better outcomes for *BRCA1/2* PV carriers, others suggested worse or similar outcomes relative to noncarriers [17–28]. Many studies indicate that *BRCA1/2*-related tumors benefit more from chemotherapy than sporadic breast cancers [29–32]. For instance, a population-based cohort study using SEER data, found lower cancer-specific mortality in *BRCA1/2* PV carriers, particularly among those with triple-negative breast cancer (TNBC) who received chemotherapy [33]. This is supported by clinical trials such as GeparSixto and GeparOcto, which demonstrated higher pathological complete response (pCR) rates in *BRCA*-related TNBC and hormone receptor-positive breast cancer, suggesting favorable chemotherapy responses [34, 35]. Additionally, *BRCA1/2* PV carriers may exhibit higher Oncotype DX recurrence scores, reflecting the high-grade nature of *BRCA*-associated breast cancer [36, 37]. This aggressive phenotype might benefit more from chemotherapy due to its heightened sensitivity to cytotoxic treatments [38]. The reduced breast cancer-specific mortality in *BRCA1/2* PV carriers could be linked to their tumors’ better response to chemotherapy and potentially more rigorous treatment regimens, including escalated chemotherapy protocols. *BRCA1/2* mutations also affect treatment efficacy by impairing DNA damage repair through homologous recombination, thereby increasing sensitivity to Poly ADP-ribose polymerase (PARP) inhibitors and platinum-based agents [39–44]. However, the overall impact of *BRCA1/2* mutations on survival outcomes in breast cancer patients remains an area of ongoing research.

The goal of this analysis was to investigate response to chemotherapy and survival outcomes in breast cancer patients with germline *BRCA1 or 2* mutations compared with those with wild-type genotypes. To assess whether *BRCA1/2* mutation has a differential impact on prognosis in breast cancer patients treated with neoadjuvant chemotherapy (NCT), while minimizing the effects of differences in treatment and clinicopathological characteristics, we utilized propensity score matching (PSM).

## Methods

### Patients and study populations

This retrospective cohort study utilized the database derived from the electronic medical records of Korea University Anam Hospital (KUAH) and Seoul National University Hospital (SNUH), including deidentified demographic and clinicopathological information. We analyzed breast cancer patients who underwent germline *BRCA1/2* testing and received NCT followed by curative resection and standard adjuvant therapy from January 2001 to January 2019. Patients with distant metastases or missing NCT response data were excluded. All patients included in this study were consecutively treated during the study period at the participating institutions. This study was approved by the Institutional Review Boards of both institutions (KUAH: 2022AN0035, 2023AN0174; SNUH: 1905-190-1038, 1507-132-689). Informed consent was waived due to the retrospective nature and minimal privacy risk. Data analysis was conducted from March to June 2024.

### Demographic and pathological variables

Invasive carcinoma was confirmed via pre-treatment core biopsy. Clinical tumor stage and lymph node (LN) metastasis status were classified according to the American Joint Committee on Cancer staging manual. Histologic type, tumor grade, and immunohistochemical (IHC) status for the estrogen receptor (ER), progesterone receptor (PgR) and human epidermal growth factor receptor 2 (HER2) were evaluated in formalin-fixed paraffin-embedded tissue. Positive ER or PgR was defined as 1% or more of stained cells with estrogen or progesterone receptor on IHC staining. HER2 positivity was defined as 3 + receptor overexpression by IHC staining and/or gene amplification detected by fluorescence/silver in situ hybridization, following American Society of Clinical Oncology and College of American Pathologists guidelines. Ki-67 indices of < 14% (KUAH) and < 10% (SNUH) were considered low, based on prior SNUH studies [45].

### Sequencing and variant analyses

Genetic mutations in *BRCA1/2* genes were analyzed as part of routine clinical testing. Germline *BRCA1/2* testing was performed on patients who met the criteria for *BRCA1/2* diagnostic testing established by the Korean Clinical Practice Guidelines for Breast Cancer [46] and was conducted upon patient request. Genomic DNA was extracted from peripheral blood samples and analyzed for germline mutations. To assess germline mutations in *BRCA1/2*, the entire coding regions and surrounding introns were included in Sanger sequencing or the next-generation sequencing methods. The pathogenicity of all detected variants was reviewed by experts from both institutions, and germline variants were classified according to the five-tier system of the American College of Medical Genetics and Genomics guidelines [47]. Variants classified as benign, likely benign or of unknown significance (VUS) were considered non-deleterious.

### Treatment

The majority of patients received a standard eight-cycle regimen consisting of anthracycline/cyclophosphamide followed by taxane (AC→T). Carboplatin was added in a subset of patients, most commonly those with triple-negative or high-risk disease. A small number of patients received PARP inhibitor–containing regimens, likely as part of investigational protocols. Anti-HER2 therapy (trastuzumab with or without pertuzumab) was administered to HER2-positive patients during and after the NCT period. After completing NCT, all patients underwent definitive breast surgery and either sentinel node biopsy or axillary LN dissection. Patients who had hormone receptor–positive disease received adjuvant endocrine therapy. Postoperative radiation therapy was administered if patients underwent breast conservation surgery or presented locally advanced disease or inflammatory breast cancer.

### Outcomes

The primary outcomes were the response to NCT and distant metastasis-free survival (DMFS) according to *BRCA1/2* mutation status. pCR was defined as on invasive cancer in the breast (ypT0/Tis) and micro- or macro-metastasis in ipsilateral axillary lymph nodes (ypN0). Distant metastasis (DM) was confirmed by imaging or pathology in distant organs or lymph nodes, excluding regional nodes or the ipsilateral/contralateral breast. DMFS was calculated from diagnosis to the first radiologic or pathologic confirmation of DM, with deaths without distant recurrence censored at the time of death. Follow-up duration was from diagnosis to the last hospital visit.

### Statistical analysis and propensity score matching (PSM)

To minimize confounding biases in comparing pCR rates and survival outcomes between patients. To reduce confounding in comparing pCR rates and survival outcomes between *BRCA1/2* mutation carriers and wild-type patients, PSM was applied. Matching variables included institution, age at diagnosis, family cancer history, menstruation status, bilateral cancer, clinical TN stage, Ki-67 levels, ER/PgR/HER2 status, and therapy regimens. Standardized mean difference (SMD) was used to evaluate balance, achieving SMD < 0.1 for all variables except ALN status (SMD = 0.131) and follow-up duration (SMD = 0.127) (Table 1). Patients were then matched 1:2 into *BRCA1/2* mutation groups and wild-type groups using the nearest neighbor matching without replacement. Categorical variables were compared using Chi-square or Fisher’s exact tests, and continuous variables with Student’s t-test. Kaplan-Meier and log-rank tests were used to assess 5-year DMFS. Statistical analyses were performed in R version 4.3.1. Two-tailed P values < 0.05 were considered significant.

**Table 1:** Patients, tumor, treatment characteristics in the matched two groups

	BRCA1/2 wild-type *n* = 170	BRCA1/2 mutation *n* = 85	Chi-square (or Fisher’s exact*) *p*-value	SMD
*BRCA1* mutation (%)		53 (62.4)		
*BRCA2* mutation (%)		32 (37.6)		
**Institution**			0.648	0.1
**Age at diagnosis** **(median [IQR])**	41.0 [36.0, 50.0]	42.0 [36.0, 51.0]	0.625	0.064
Follow-up duration (median [IQR])	46.0 [29.0, 79.8]	50.0 [33.00, 81.0]	0.321	0.127
**Family history_any organ cancer (%)**			0.892	0.063
No	40 (23.5)	22 (25.9)		
Yes	114 (67.1)	56 (65.9)		
Unknown	16 (9.4)	7 (8.2)		
Family history_breast cancer (%)			0.352	0.197
No	41 (24.1)	14 (16.5)		
Yes	73 (42.9)	42 (49.4)		
Unknown	56 (32.9)	29 (34.1)		
Family history_ovarian cancer (%)			*0.004*	0.417
No	105 (61.8)	41 (48.2)		
Yes	9 (5.3)	15 (17.6)		
Unknown	56 (32.9)	29 (34.1)		
Family history_pancreas cancer (%)			0.965*	0.042
No	109 (64.1)	54 (63.5)		
Yes	5 (2.9)	2 (2.4)		
Unknown	56 (32.9)	29 (34.1)		
Family history_prostate cancer (%)			0.852*	0.069
No	113 (66.5)	55 (64.7)		
Yes	1 (0.6)	1 (1.2)		
Unknown	56 (32.9)	29 (34.1)		
**Menstruation status (%)**			0.878	0.067
Pre-menopausal	117 (68.8)	56 (65.9)		
Post-menopausal	34 (20.0)	18 (21.2)		
Unknown	19 (11.2)	11 (12.9)		
**Bilateral cancer (%)**	14 (8.2)	7 (8.2)	1.000	< 0.001
Histology (%)			0.800*	0.19
Ductal	165 (97.1)	84 (98.8)		
Lobular	3 (1.8)	0 (0.0)		
Other	2 (1.2)	1 (1.2)		
**Tumor size (cT) (%)**			0.857*	0.07
≤ 5 cm(cT1,2)	123 (72.4)	61 (71.8)		
> 5 cm(cT3,4)	43 (25.3)	21 (24.7)		
Unknown	4 (2.4)	3 (3.5)		
**Axillary lymph node metastasis(cN) (%)**			0.738*	0.131
No (N0)	29 (17.1)	17 (20.0)		
Yes (N+)	140 (82.4)	68 (80.0)		
Unknown	1 (0.6)	0 (0.0)		
**Ki-67 (%)**			0.965	0.035
High	115 (67.6)	57 (67.1)		
Low	33 (19.4)	16 (18.8)		
Unknown	22 (12.9)	12 (14.1)		
**ER-positive (%)**	100 (58.8)	49 (57.6)	0.964	0.024
**PgR-positive (%)**	65 (38.2)	30 (35.3)	0.749	0.061
**HER2 status (%)**			1.000*	0.021
Negative	155 (91.2)	78 (91.8)		
Positive	15 (8.8)	15 (8.8)		
TNBC (%)	66 (38.8)	32 (37.6)	0.892*	0.024
**NCT regimen_Known (%)**	170 (100.0)	85 (100.0)	1.000*	< 0.001
**NCT regimen (AC followed by T) (%)**			1.000*	< 0.001
No	14 (8.2)	7 (8.2)		
Yes	156 (91.8)	78 (91.8)		
**NCT regimen (carboplatin-containing) (%)**			1.000*	< 0.001
No	158 (92.9)	79 (92.9)		
Yes	12 (7.1)	6 (7.1)		
**NCT regimen (PARP inhibitor-containing) (%)**			0.689*	0.07
No	166 (97.6)	82 (96.5)		
Yes	4 (2.4)	3 (3.5)		
**Trastuzumab use (%)**			1.000*	< 0.001
No	160 (94.1)	80 (94.1)		
Yes	10 (5.9)	5 (5.9)		
Breast operation (%)			*0.046*	0.285
Mastectomy	76 (44.7)	50 (58.8)		
Conservation	94 (55.3)	35 (41.2)		
Axilla operation (%)			0.965	0.024
SLNB alone	92 (54.1)	47 (55.3)		
ALND	78 (45.9)	38 (44.7)		

SMD; standardized mean difference (SMD < 0.1 suggests that the groups are well-balanced concerning the characteristic being measured.), IQR; interquartile range, ER; estrogen receptor, PgR; progesterone receptors, HER2; human epidermal growth factor receptor 2, TNBC; triple-negative breast cancer, NCT; neoadjuvant chemotherapy, AC; anthracycline, T; taxane, SLNB; sentinel lymph node biopsy, ALND; axillary lymph node dissection, *; Fisher’s exact test, Bold text; variables to match

## Results

### Patient, tumor and treatment characteristics

Of the 411 patients included, 86 (20.9%) had *BRCA1/2* mutations, including 54 *BRCA1* and 32 *BRCA2* pathogenic variants (PVs). Before matching, patients in the *BRCA1/2* mutation group were more likely to have family history of ovarian cancer (*P* < 0.001), HER2-negative disease (*P* < 0.001), receive less anti-HER2 therapy (*P =* 0.001), and undergo more anthracycline followed by taxane regimens (*P =* 0.005) and carboplatin-containing regimens (*P =* 0.033) compared to those in the wild-type group. Mean age at diagnosis, menstruation status, clinical TN stage and ER/PgR status did not show significant differences between the two groups (Supplementary Table 1). After matching, *BRCA1/2* mutation and wild-type groups included 170 and 85 patients, respectively. There were no differences between the two matched groups in demographic characteristics except for family history of ovarian cancer and type of breast operation. Family history of ovarian cancer (*P* = 0.004, SMD = 0.417) and mastectomy rates (*P* = 0.046, SMD = 0.285) were higher in mutation carriers. Other demographic, pathological, and neoadjuvant systemic therapy features showed no significant differences (Table 1).

### Response to chemotherapy relative to *BRCA1/2* mutation status in matched cohort

After matching, pCR rate was 26.5% (45/170) for wild-type patients and 40.0% (34/85) for *BRCA1/2* PV carriers (*BRCA1/2* mutation vs. wild-type, OR = 1.85, 95% CI = 1.07–3.22, *P =* 0.029). Among the subgroups, 26 (49.1%) patients of the 53 matched *BRCA1* PV carriers achieved pCR (*BRCA1* mutation vs. wild-type, OR = 2.71, 95% CI = 1.45–5.05, *P =* 0.002), whereas 8 (25.0%) of the 32 matched *BRCA2* PV carriers achieved pCR (*BRCA2* mutation vs. wild-type, OR = 0.71, 95% CI = 0.31–1.67, *P =* 0.436) (Table 2).

### Distant metastasis free survival (DMFS) and overall survival (OS) relative to *BRCA1/2* mutation status and pCR in matched cohort

After matching, the median follow-up period was 46.0 [29.00-79.75] months for the wild-type group and 50.0 [33.0–81.0] months for the mutation carrier group (*P =* 0.321). During this period, there were a total of 35 cases of DM and 25 cases of all-cause death observed in both matched groups. Both pCR (*P* = 0.015) and *BRCA1/2* mutations (*P* = 0.006) were independent favorable prognostic factors for DM in the matched group (Table 2). Regardless of *BRCA1/2* mutations, patients with pCR had significantly lower rates of DM (1 case (1.3%) vs. 34 cases (19.3%), pCR vs. non-pCR, *P =* 0.004) and all-cause death compared to those without pCR (2 cases (2.5%) vs. 23 cases (12.6%), pCR vs. non-pCR, *P =* 0.019). In the subset of 176 patients who did not achieve pCR, 5-year DM rate was lower in *BRCA1/2* PV carriers than in noncarriers (4 cases (7.8%) vs. 30 cases (24.0%), *BRCA1/2* mutation vs. wild-type, *P =* 0.02). However, the 5-year all-cause death rate did not differ between *BRCA1/2* PV carriers and noncarriers in patients without pCR (4 cases (7.8%) vs. 19 cases (15.2%), *BRCA1/2* mutation vs. wild-type, *P =* 0.197) (Table 2).

**Table 2:** Logistic regression analysis for pCR rates by *BRCA1/2* mutation status and survival outcomes in the matched two groups

	Odds Ratio (95%CI)	*P* value
pCR rate by *BRCA1/2* mutation status		
*BRCA1/2*: Mutation vs. Wild-type	1.85 (1.07,3.22)	0.029
*BRCA1*: Mutation vs. Wild-type	2.71 (1.45,5.05)	0.002
*BRCA2*: Mutation vs. Wild-type	0.71 (0.31,1.67)	0.436
Distant metastasis events (*n* = 35) by *BRCA1/2* mutation status and pCR		
pCR: Yes vs. No	0.06 (0.01,0.45)	0.006
*BRCA1/2*: Mutation vs. Wild-type	0.26 (0.09,0.76)	0.015
*BRCA1*: Mutation vs. Wild-type	0.24 (0.07,0.82)	0.037
*BRCA2*: Mutation vs. Wild-type	0.13 (0.02,0.98)	0.042
All-cause death events (*n* = 25) by *BRCA1/2* mutation status and pCR		
pCR: Yes vs. No	0.19 (0.04,0.81)	0.025
*BRCA1/2*: Mutation vs. Wild-type	0.54 (0.19,1.52)	0.244
Subgroup analysis in patients with non-pCR (*n* = 176)		
Distant metastasis events (*n* = 34) by *BRCA1/2* mutation status		
*BRCA1/2*: Mutation vs. Wild-type	0.27 (0.09,0.81)	0.02
All-cause death events (*n* = 23) by *BRCA1/2* mutation status		
*BRCA1/2*: Mutation vs. Wild-type	0.47 (0.15,1.47)	0.197

**Fig. 1:**
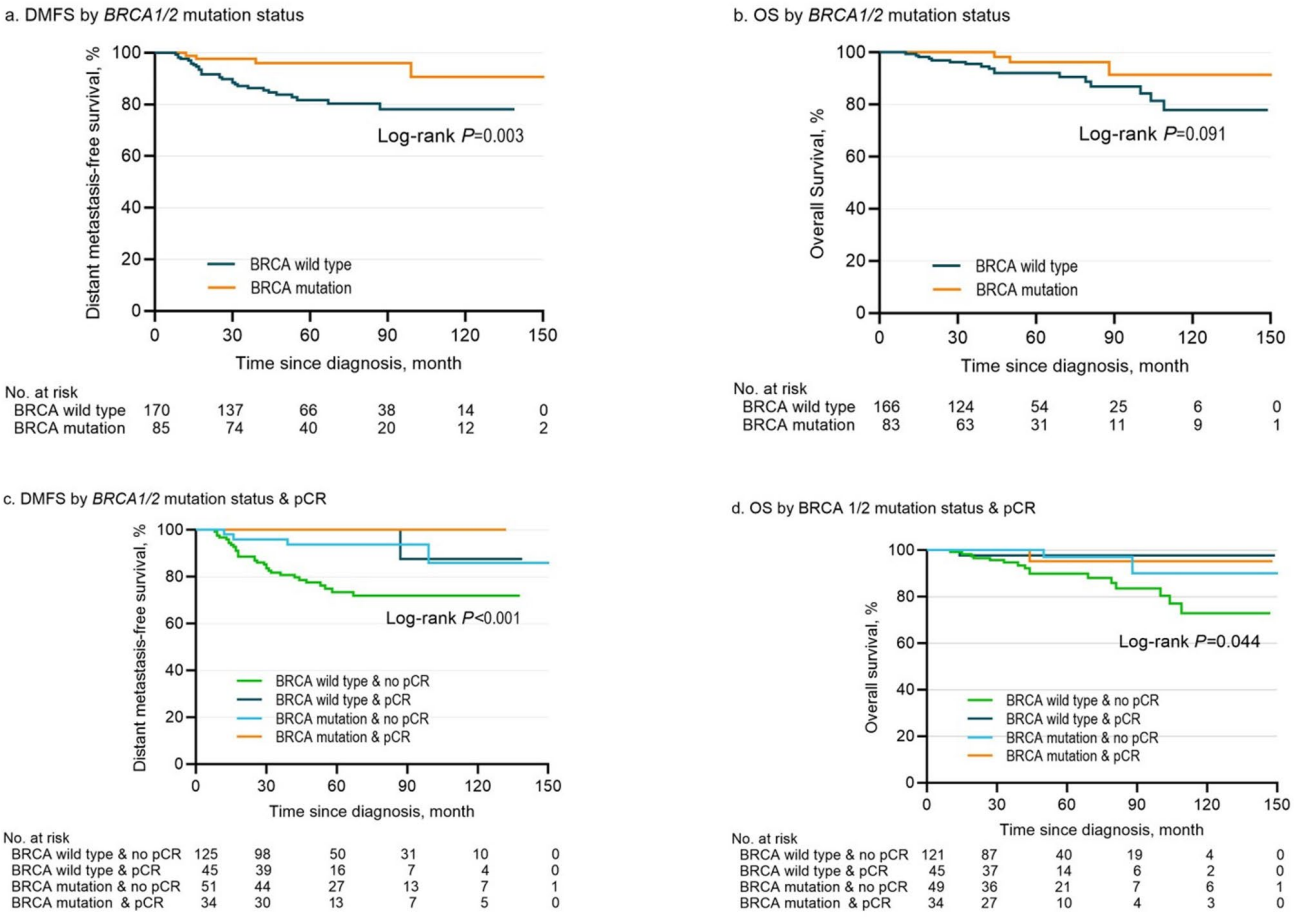
Kaplan-meier analysis of distant metastasis-free survival and overall survival by *BRCA 1/2* status and pCR

In both matched groups, the 5-year DMFS was significantly associated with pCR (*P =* 0.001) (Supplementary Fig. 1) and *BRCA1/2* mutation status (*P =* 0.003) (Fig. 1). While pCR was significantly related to OS (*P =* 0.041) (Supplementary Fig. 1), the difference in OS between *BRCA1/2* PV carriers and noncarriers did not reach statistical significance (*P =* 0.091) (Fig. 1). Patients achieving pCR demonstrated prolonged DMFS and OS, irrespective of their *BRCA 1/2* mutation status. Among the 176 patients who did not achieve pCR, those with *BRCA1/2* PV carriers had a significantly better DMFS compared to wild-type patients (hazard ratio (HR) = 0.27, 95% confidence interval (CI) = 0.09–0.81, *P =* 0.02). However, there was no significant difference in OS between *BRCA1/2* PV carriers and noncarriers (HR = 0.47, 95% CI = 0.15–1.47, *P =* 0.197) (Fig. 1; Table 3). Additionally, the Cox proportional hazards model indicated that pCR (HR = 0.08, 95% CI = 0.08–0.08, *P =* 0.01), and *BRCA1/2* PV carriers (HR = 0.09, 95% CI = 0.09–0.09, *P =* 0.016) were independently associated with longer DMFS. However, this significance was not observed for OS in the matched cohorts (Table 3).

**Table 3:** Distant metastasis free survival and overall survival by *BRCA1/2* status and pCR in the matched two groups (Cox proportional analysis)

	Odds Ratio (95%CI)	*P* value
Distant metastasis free survival		
*BRCA1/2*: Mutation vs. Wild-type	0.09 (0.09,0.09)	0.016
pCR: Yes vs. No	0.08 (0.08,0.08)	0.01
No. of observations	255	
No. of events	35	
Overall survival		
*BRCA1/2*: Mutation vs. Wild-type	0.23 (0.17,0.3)	0.142
pCR: Yes vs. No	0.17 (0.15,0.19)	0.075
No. of observations	249*	
No. of events	19	
Subgroup analysis in patients with non-pCR (*n* = 176)		
Distant metastasis events (*n* = 34) by *BRCA1/2* mutation status		
*BRCA1/2*: Mutation vs. No mutation	0.1 (0.1,0.1)	0.022
No. of observations	176	
No. of events	34	
All-cause death events (*n* = 23) by *BRCA1/2* mutation status		
*BRCA1/2*: Mutation vs. No mutation	0.2 (0.16,0.25)	0.107
No. of observations	170*	
No. of events	17	

* Out of 25 death events, 6 cases were excluded due to unavailable date of death information.

## Discussion

Beyond genetic risk, *BRCA1/2* mutations appear to influence how tumors respond to systemic therapy, with implications for prognosis. In a retrospective study of 317 patients published in 2011, Arun et al. [48] reported that *BRCA1* mutation carriers had markedly higher pCR rates (46%) than BRCA2 carriers (13%) or noncarriers (22%), with pCR translating to better survival only among *BRCA1* carriers. Supporting this finding, a study based on clinical data from a German breast cancer reported a pCR rate of 54.3% in *BRCA1/2* mutation carriers versus 22.6% in noncarriers (adjusted odds ratio [OR] = 2.48, 95% confidence interval [CI] 1.26–4.91), with pCR emerging as the strongest predictor of both disease-free and overall survival, independent of *BRCA* 1/2 mutation status [31]. Our matched cohort analysis confirmed this trend and further contributed additional evidence regarding *BRCA* mutation–associated prognosis. Among patients who did not achieve pCR, *BRCA1/2* mutation carriers experienced fewer distant metastases compared to noncarriers, indicating a potential survival benefit in this subgroup. Conversely, prognosis was uniformly favorable for those who did achieve pCR, irrespective of *BRCA* mutation status, with only one distant event observed in the entire matched pCR cohort. These findings reinforce the prognostic significance of pCR and suggest that *BRCA*-associated tumor biology may influence outcomes beyond pathologic response.

In terms of *BRCA*-stratified prospective data, the TNT trial demonstrated that *BRCA1/2* mutation carriers achieved a pCR rate of 66.7% without carboplatin, which was higher than that of non-carriers, whose pCR rates were 36.4% without and 55.0% with carboplatin [41]. In contrast, the BRIGHTNESS trial evaluated *BRCA1/2* mutation carriers and noncarriers matched by treatment arm, lymph node status, and age, and found no significant difference in pCR rates between the groups. Specifically, the odds of achieving pCR were not higher in *BRCA* mutation carriers receiving standard NCT with carboplatin (OR 0.24, 95% CI 0.04–1.24, *P* = 0.09) or with carboplatin/veliparib (OR 0.44, 95% CI 0.10–1.84, *P* = 0.26) compared to noncarriers, suggesting no additional benefit from the inclusion of platinum or PARP inhibitors based on *BRCA* status [32]. Efforts to identify chemotherapy regimens that may offer greater benefit specifically for *BRCA* mutation carriers, particularly those involving optimized backbones or additive agents, have continued through prospective trials. The INFORM trial, a randomized phase II study, compared cisplatin and doxorubicin-cyclophosphamide in *BRCA 1/2* carriers with HER2-negative breast cancer and found no significant difference in pCR or RCB 0/1 between the two arms [43]. Similarly, the other clinical trials demonstrated no additional pCR benefit with the addition of platinum agents for *BRCA* 1/2 mutation carriers diagnosed with TNBC, despite platinum-related improvements observed in noncarriers [32, 34]. In our cohort, multivariate logistic regression using the unmatched dataset (*N* = 411) showed that *BRCA1/2* mutation status, tumor size > 5 cm, ER-negative tumor, and the use of carboplatin-containing regimens were independently associated with achieving pCR. Among them, the number of patients who received carboplatin was relatively small (*N* = 62), yet its use was associated with an increased likelihood of achieving pCR (adjusted OR = 3.27, 95% CI: 1.28–8.33, *P* = 0.013) (Supplementary Table 2). Similarly, although PARP inhibitors were administered to only seven patients (Supplementary Table 1), six of them achieved pCR, suggesting a strong potential effect despite very small sample size. Distant recurrence, on the other hand, was more frequently observed in *BRCA1/2* wild-type patients with non-pCR. The chemotherapy regimen, including carboplatin, was associated with an increased likelihood of pCR but did not appear to influence distant recurrence (Supplementary Table 2).

While most of the existing literature has focused on *BRCA1* mutations and TNBC, data remain limited for *BRCA2* carriers and hormone receptor–positive subtypes. In our matched ER-positive cohort (*n* = 149), *BRCA1/2* mutation status was not associated with a significantly higher pCR rate (OR = 1.36, 95% CI = 0.61–3.01, *P* = 0.451), but carriers demonstrated a significantly lower risk of DM events (HR = 0.13, 95% CI = 0.12–0.14, *P* = 0.041), as shown in Table 4. Although the sample size was limited, the observed DM events and DMFS differences were statistically significant, suggesting that the prognostic impact of *BRCA* mutations may extend beyond TNBC and beyond the achievement of pCR, warranting further investigation in ER-positive disease. This contrasts with the findings of Talhouet et al. [27], who reported that *BRCA1/2* mutations were associated with improved survival only in patients with TNBC, with no survival benefit observed in non-TNBC subtypes.

**Table 4:** pCR by BRCA1/2 status and DMFS by BRCA1/2 status and pCR in the matched two groups with ER-positive breast cancer (*n* = 149)

	Odds Ratio (95%CI)	*P* value
pCR rate by *BRCA1/2* mutation status (Logistic regression analysis)		
*BRCA1/2*: Mutation vs. Wild-type	1.36 (0.61,3.01)	0.451
*BRCA1*: Mutation vs. Wild-type	2.82 (1.09,7.35)	0.033
*BRCA2*: Mutation vs. Wild-type	0.53 (0.17,1.67)	0.279
Distant metastasis events (*n* = 23) by *BRCA1/2* mutation status and pCR (Logistic regression analysis)		
*BRCA1/2*: Mutation vs. Wild-type	0.27 (0.07,0.97)	0.044
pCR: Yes vs. No	0.13 (0.02,1.03)	0.053
Distant metastasis-free survival by *BRCA1/2* mutation status and pCR (Cox proportional analysis)		
*BRCA1/2*: Mutation vs. No mutation	0.13 (0.12,0.14)	0.041
pCR: Yes vs. No	0.15 (0.14,0.17)	0.06
No. of observations	149	
No. of events	23	

This study has several limitations. First, although we attempted to reduce the impact of selection bias using PSM to control for key confounders, the retrospective nature of the study remains a source of potential bias. Second, the relatively short follow-up period may not be sufficient to fully capture long-term outcomes. The POSH (Prospective Study of Outcomes in Sporadic and Hereditary breast cancer) trial [26] indicated that *BRCA1/2* PV carriers with TNBC might experience an early survival advantage compared to noncarriers within the first 2 years after diagnosis; however, this benefit diminished over time, resulting in similar long-term outcomes. Additionally, ER-positive tumors are known to have higher rates of late recurrence beyond five years, reinforcing the need for extended follow-up in this population. Third, our dataset did not account for important confounding variables such as prophylactic mastectomy or salpingo-oophorectomy, which could influence survival outcomes. Lastly, the small number of patients, particularly in subgroup analyses, restricted the ability to draw definitive conclusions and highlights the necessity for larger studies with comprehensive clinical data, including stratification by tumor subtype and distinction between *BRCA1* and *BRCA2* mutation carriers, to validate and extend these findings.

## Conclusions

In conclusion, our study employed PSM to evaluate the impact of *BRCA1/2* mutations on pCR rates and survival outcomes in breast cancer. The results provide valuable insights into the association between *BRCA1/2* mutations and both increased pCR rates and prolonged DMFS. These findings can help tailor treatment approaches and inform patient discussions about prognosis and adjuvant treatment options following NCT.

## Key points

**Question**: How do germline *BRCA1/2* mutations influence neoadjuvant chemotherapy response and survival outcomes in breast cancer patients?

**Findings**: *BRCA1/2* PV carriers had a higher pCR rate (40.0% vs. 26.5%, OR = 1.85, 95% CI = 1.07–3.22, *P =* 0.029) and a lower 5-year distant metastasis rate (4.7% vs. 18.2%, OR = 0.22, 95% CI: 0.08–0.65, *P =* 0.006). In patients who did not achieve pCR, *BRCA1/2* PV carriers showed better distant metastasis-free survival (HR = 0.27, 95% CI = 0.09–0.81, *P =* 0.02), though overall survival differences were not significant.

**Meaning**: Germline *BRCA1/2* mutations are linked to improved chemotherapy response and survival, suggesting their role in treatment planning.

## Electronic supplementary material

Below is the link to the electronic supplementary material.

Supplementary Material 1

## Data Availability

The datasets generated and/or analyzed during the current study are not publicly available due to institutional restrictions, but are available from the corresponding author on reasonable request.
