# Supplementary material for: Impact of germline BRCA1/2 mutations on response to neoadjuvant systemic therapy and prognosis in breast cancer: a propensity score matched cohort study
Source: Breast Cancer Res. 2025 May 22;27:89. doi: 10.1186/s13058-025-02041-6 (PMC12096599; doi:10.1186/s13058-025-02041-6)
Supplement: Supplementary file 1 — Supplementary Material 1 [file 13058_2025_2041_MOESM1_ESM.docx]

Supplementary Table 1 Patients, tumor, treatment characteristics in the unmatched cohort

|  | *BRCA1/2* wild-type  n= 325 | *BRCA1/2* mutation  n= 86 | Chi-square (or Fisher’s exact) *p*-value | SMD |
| --- | --- | --- | --- | --- |
| *BRCA1* mutation (%) |  | 54 (62.8) |  |  |
| *BRCA2* mutation (%) |  | 32 (37.2) |  |  |
| Institution |  |  | 0.099 | 0.25 |
| Age at diagnosis  (median [IQR]) | 41.0  [36.0, 49.0] | 42.0  [36.00, 50.5] | 0.542 | 0.09 |
| Follow-up duration  (median [IQR]) | 47.0  [31.0, 78.0] | 50.5  [33.0, 81.0] | 0.389 | 0.118 |
| Family history_any organ cancer (%) | | | 0.82 | 0.077 |
| No | 98 (30.2) | 23 (26.7) |  |  |
| Yes | 203 (62.5) | 56 (65.1) |  |  |
| Unknown | 24 (7.4) | 7 (8.1) |  |  |
| Family history_breast cancer (%) | | | 0.647 | 0.113 |
| No | 62 (19.1) | 14 (16.3) |  |  |
| Yes | 141 (43.4) | 42 (48.8) |  |  |
| Unknown | 122 (37.5) | 30 (34.9) |  |  |
| Family history_ovarian cancer (%) | | | <0.001 | 0.436 |
| No | 189 (58.2) | 41 (47.7) |  |  |
| Yes | 14 (4.3) | 15 (17.4) |  |  |
| Unknown | 122 (37.5) | 30 (34.9) |  |  |
| Family history_pancreas cancer (%) | | | 0.880* | 0.056 |
| No | 196 (60.3) | 54 (62.8) |  |  |
| Yes | 7 (2.2) | 2 (2.3) |  |  |
| Unknown | 122 (37.5) | 30 (34.9) |  |  |
| Family history_prostate cancer (%) | | | 0.578* | 0.078 |
| No | 201 (61.8) | 55 (64.0) |  |  |
| Yes | 2 (0.6) | 1 (1.2) |  |  |
| Unknown | 122 (37.5) | 30 (34.9) |  |  |
| Menstruation status (%) | | | 0.411 | 0.159 |
| Pre-menopausal | 239 (73.5) | 57 (66.3) |  |  |
| Post-menopausal | 53 (16.3) | 18 (20.9) |  |  |
| Unknown | 33 (10.2) | 11 (12.8) |  |  |
| Bilateral cancer (%) | 30 (9.2) | 8 (9.3) | 1.000 | 0.002 |
| Histology (%) | | | 0.645* | 0.197 |
| Ductal | 314 (96.6) | 85 (98.8) |  |  |
| Lobular | 6 (1.8) | 0 (0.0) |  |  |
| Other | 5 (1.5) | 1 (1.2) |  |  |
| Tumor size (cT) (%) | | | 0.608* | 0.093 |
| ≤5cm(cT1,2) | 244 (75.1) | 62 (72.1) |  |  |
| >5cm(cT3,4) | 74 (22.8) | 21 (24.4) |  |  |
| Unknown | 7 (2.2) | 3 (3.5) |  |  |
| Axillary lymph node metastasis(cN) (%) | | | 0.155* | 0.255 |
| No (N0) | 40 (12.3) | 17 (19.8) |  |  |
| Yes (N+) | 281 (86.5) | 69 (80.2) |  |  |
| Unknown | 4 (1.2) | 0 (0.0) |  |  |
| Ki-67 (%) | | | 0.236 | 0.213 |
| High | 186 (57.2) | 57 (66.3) |  |  |
| Low | 88 (27.1) | 16 (18.6) |  |  |
| Unknown | 51 (15.7) | 13 (15.1) |  |  |
| ER-positive (%) | 178 (56.0) | 49 (57.6) | 0.878 | 0.034 |
| PgR-positive (%) | 130 (41.0) | 30 (34.9) | 0.365 | 0.126 |
| HER2 status (%) | | | <0.001* | 0.554 |
| Negative | 233 (71.7) | 79 (91.9) |  |  |
| Positive | 83 (25.5) | 7 (8.1) |  |  |
| Unkown | 9 (2.8) | 0 (0.0) |  |  |
| TNBC (%) | | | 0.472* | 0.177 |
| No | 214 (65.8) | 53 (61.6) |  |  |
| Yes | 101 (31.1) | 32 (37.2) |  |  |
| Unknown | 10 (3.1) | 1 (1.2) |  |  |
| NCT regimen (%) |  |  | 1.000* | 0.137 |
| Known | 322 (99.1) | 86 (100.0) |  |  |
| Unknown | 3 (0.9) | 0 (0.0) |  |  |
| NCT regimen (AC followed by T) (%) | | | 0.005* | 0.427 |
| No | 72 (22.2) | 7 (8.1) |  |  |
| Yes | 250 (76.9) | 79 (91.9) |  |  |
| Unknown | 3 (0.9) | 0 (0.0) |  |  |
| NCT regimen (carboplatin-containing) (%) | | | 0.033* | 0.351 |
| No | 266 (81.8) | 80 (93.0) |  |  |
| Yes | 56 (17.2) | 6 (7.0) |  |  |
| Unknown | 3 (0.9) | 0 (0.0) |  |  |
| NCT regimen (PARP inhibitor-containing) (%) | | | 0.257* | 0.202 |
| No | 318 (97.8) | 83 (96.5) |  |  |
| Yes | 4 (1.2) | 3 (3.5) |  |  |
| Unknown | 3 (0.9) | 0 (0.0) |  |  |
| Trastuzumab use (%) | | | 0.001* | 0.496 |
| No | 252 (77.5) | 81 (94.2) |  |  |
| Yes | 70 (21.5) | 5 (5.8) |  |  |
| Unknown | 3 (0.9) | 0 (0.0) |  |  |
| Breast operation (%) | | | 0.121 | 0.204 |
| Mastectomy | 156 (48.0) | 50 (58.1) |  |  |
| Conservation | 169 (52.0) | 36 (41.9) |  |  |
| Axilla operation (%) | | | 0.647 | 0.07 |
| SLNB alone | 170 (52.3) | 48 (55.8) |  |  |
| ALND | 155 (47.7) | 38 (44.2) |  |  |

SMD; standardized mean difference (SMD < 0.1 suggests that the groups are well-balanced concerning the characteristic being measured.), IQR; interquartile range, ER; estrogen receptor, PgR; progesterone receptors, HER2; human epidermal growth factor receptor 2, TNBC; triple-negative breast cancer, NCT; neoadjuvant chemotherapy, AC; anthracycline plus cycholophosphamide, T; taxane, SLNB; sentinel lymph node biopsy, ALND; axillary lymph node dissection, *; Fisher’s exact test, Bold text; variables to match

Supplementary Table 2 Logistic regression analyses identifying predictors of pathological complete response and distant metastasis events in the unmatched cohort (N = 411)

|  | Logistic regression predicting pCR | | | | Logistic regression predicting distant metastasis events | | | |
| --- | --- | --- | --- | --- | --- | --- | --- | --- |
|  | Univariate analysis | | Multivariate analysis | | Univariate analysis | | Multivariate analysis | |
|  | Odds Ratio (95%CI) | *P* value | Odds Ratio (95%CI) | *P* value | Odds Ratio (95%CI) | *P* value | Odds Ratio (95%CI) | *P* value |
| *BRCA1/2*: Mutation vs Wild-type | 1.71 (1.04,2.8) | 0.034 | 2.3 (1.31,4.03) | 0.004 | 0.3 (0.11,0.87) | 0.026 | 0.3 (0.1,0.88) | 0.029 |
| tumor size (cT): reference = ≤ 5cm (cT1,2) | | | |  |  |  |  |  |
| >5cm (cT3,4) | 0.35 (0.19,0.64) | < 0.001 | 0.36 (0.19,0.67) | 0.002 | 1.87 (0.98,3.54) | 0.056 | 1.54 (0.78,3.05) | 0.211 |
| Unknown | 0.81 (0.2,3.19) | 0.762 | 0.84 (0.18,3.81) | 0.818 | 0 (0,Inf) | 0.985 | 0 (0,Inf) | 0.989 |
| Axillary lymph node metastasis (cN): reference = No (N0) | | | N/A |  |  |  | N/A |  |
| Yes (N+) | 0.75 (0.42,1.36) | 0.341 |  |  | 2.72 (0.82,9.07) | 0.103 |  |  |
| Unknown | 5.55 (0.54,56.91) | 0.149 |  |  | 0 (0,Inf) | 0.986 |  |  |
| ER: Positive vs Negative | 0.35 (0.23,0.55) | < 0.001 | 0.37 (0.23,0.59) | < 0.001 | 1.1 (0.6,2.02) | 0.765 | N/A |  |
| HER2 status: reference=Negative | | |  |  |  |  |  |  |
| Positive | 2.08 (1.28,3.38) | 0.003 | 1.61 (0.79,3.29) | 0.19 | 0.13 (0.03,0.55) | 0.006 | 0.18 (0.04,0.88) | 0.034 |
| Unknown | 0.34 (0.04,2.75) | 0.312 | 2.37 (0.05,103.59) | 0.655 | 0.72 (0.09,5.92) | 0.762 | 0.51 (0.06,4.36) | 0.535 |
| ki-67: reference=High | |  |  |  |  |  | N/A |  |
| Low | 0.59 (0.35,0.99) | 0.046 | 0.59 (0.32,1.07) | 0.083 | 0.62 (0.29,1.36) | 0.236 |  |  |
| Unknown | 0.52 (0.27,1) | 0.048 | 0.74 (0.37,1.51) | 0.415 | 0.94 (0.41,2.16) | 0.888 |  |  |
| NCT regimen (AC followed by T): reference=No | | | |  |  |  |  |  |
| Yes | 0.44 (0.26,0.72) | 0.001 | 0.98 (0.41,2.32) | 0.958 | 4.01 (1.21,13.27) | 0.023 | 1.59 (0.41,6.18) | 0.5 |
| Unknown | 0 (0,Inf) | 0.977 | 0 (0,Inf) | 0.982 | 12.67 (0.88,181.66) | 0.062 | 4.79 (0.22,105.61) | 0.321 |
| NCT regimen (carboplatin-containing): reference=No | | | |  |  |  | N/A |  |
| Yes | 3.45 (1.98,6.02) | < 0.001 | 3.27 (1.28,8.33) | 0.013 | 0 (0,Inf) | 0.984 |  |  |
| Unknown | 0 (0,Inf) | 0.986 | NA (NA,NA) |  | 3.1 (0.28,34.9) | 0.359 |  |  |
| pCR | N/A |  | N/A |  | 0.04 (0.01,0.3) | 0.002 | 0.06 (0.01,0.43) | 0.006 |

CI; confidence interval, N/A: not applicable, ER; estrogen receptor, HER2; human epidermal growth factor receptor 2, NCT; neoadjuvant chemotherapy, AC; anthracycline plus cyclophosphamide, T; taxane, pCR: pathological complete response

Legend


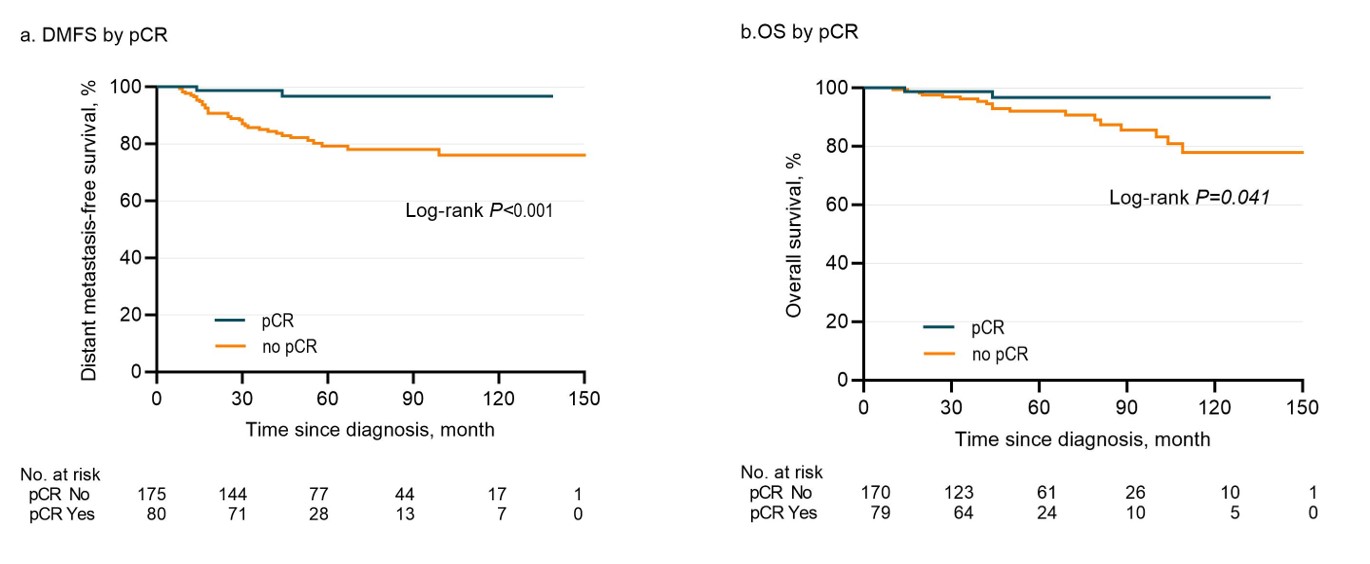


Supplementary Figure 1 Kaplan-Meier Analysis of Distant Metastasis-Free Survival and Overall Survival by pCR in both matched cohorts
